# Long-term cognitive, psychosocial, and neurovascular complications of unilateral head and neck irradiation in young to middle-aged adults

**DOI:** 10.1186/s12885-022-09295-9

**Published:** 2022-03-05

**Authors:** Judith T. Pruijssen, Ashwin Wenmakers, Roy P. C. Kessels, Vitoria Piai, Frederick J. A. Meijer, Sjoert A. H. Pegge, Jacqueline J. Loonen, Anil M. Tuladhar, Hendrik H. G. Hansen, Johannes H. A. M. Kaanders, Joyce Wilbers

**Affiliations:** 1 Medical Ultrasound Imaging Center (MUSIC), Department of Medical Imaging/Radiology, Radboud Institute Health Sciences, Radboud University Medical Center, Geert Grooteplein Zuid 10, 6525 GA Nijmegen, the Netherlands; 2 Department of Radiation Oncology, Radboud University Medical Center, Nijmegen, the Netherlands; 3 Department of Medical Psychology, Radboud University Medical Center, Nijmegen, the Netherlands; 4 Donders Institute for Brain, Cognition and Behaviour, Centre for Cognition, Radboud University, Nijmegen, the Netherlands; 5 Vincent Van Gogh Institute for Psychiatry, Venray, The Netherlands; 6 Donders Institute for Brain, Cognition and Behaviour, Centre for Medical Neuroscience, Radboud University Medical Center, Nijmegen, the Netherlands; 7 Department of Imaging/Radiology, Radboud University Medical Center, Nijmegen, the Netherlands; 8 Center of Expertise for Cancer Survivorship, Radboud University Medical Center, Nijmegen, the Netherlands; 9 Department of Neurology, Radboud University Medical Center, Nijmegen, the Netherlands

**Keywords:** Head and neck cancer, Radiotherapy, Cognition, Fatigue, Quality of life

## Abstract

**Background:**

With a growing, younger population of head and neck cancer survivors, attention to long-term side-effects of prior, often radiotherapeutic, treatment is warranted. Therefore, we studied the long-term cognitive effects in young adult patients irradiated for head and neck neoplasms (HNN).

**Methods:**

Young to middle-aged adults with HNN (aged 18-40 years) and treated with unilateral neck irradiation ≥ 5 years before inclusion underwent cardiovascular risk and neuropsychological assessments and answered validated questionnaires regarding subjective cognitive complaints, fatigue, depression, quality of life, and cancer-specific distress. Additionally, magnetic resonance imaging (MRI) of the brain was performed to assess white matter hyperintensities (WMH), infarctions, and atrophy.

**Results:**

Twenty-nine patients (aged 24–61, 13 men) median 9.2 [7.3–12.9] years post-treatment were included. HNN patients performed worse in episodic memory (Z-score = -1.16 [-1.58–0.34], p < 0.001) and reported more fatigue symptoms (Z-score = 1.75 [1.21–2.00], p < 0.001) compared to normative data. Furthermore, patients had a high level of fear of tumor recurrence (13 patients [44.8%]) and a heightened speech handicap index (13 patients [44.8%]). Only a small number of neurovascular lesions were found (3 infarctions in 2 patients and 0.11 [0.00–0.40] mL WMH), unrelated to the irradiated side. Cognitive impairment was not associated with WMH, brain atrophy, fatigue, or subjective speech problems.

**Conclusions:**

HNN patients showed impairments in episodic memory and an increased level of fatigue ≥ 5 years after radiotherapy compared to normative data. Cognitive impairments could not be explained by WMH or brain atrophy on brain MRI or psychological factors.

**Trial registration:**

Clinicaltrials.gov (https://clinicaltrials.gov/ct2/show/NCT04257968).

**Supplementary Information:**

The online version contains supplementary material available at 10.1186/s12885-022-09295-9.

## Background

Head and neck cancer (HNC) survival has increased over the past decades due to improved diagnostic and therapeutic modalities, such as modern diagnostic imaging, improved radiotherapy planning, and concurrent chemo-, immuno-, and radiotherapy [1–3]. A growing population of HNC survivors requires insight into long-term side-effects of prior radiotherapy [4, 5]. Although neurocognitive side-effects in other cancer types such as breast, colorectal, and prostate cancer are well established [6], little is known about these side-effects in HNC survivors.

HNC is a heterogeneous disease, including tumors of different histologies and at different sites such as carcinomas of the oral cavity, larynx, and (naso)pharynx but also salivary gland tumors. All these subtypes vary regarding treatment regimens and side-effects. However, cognitive decline within 2 years after treatment has been shown in various HNC populations in multiple small prospective studies, affecting memory, verbal fluency, motor function, and speed of information processing [7–14]. This cognitive decline seemed to persist > 5 years after treatment [15–17]. Concerning subjective complaints, memory problems, fatigue, anxiety, reduced quality of life, and depressive symptoms are frequently reported in HNC survivors [7, 9, 10, 14, 16–19].

Limited literature exists on the correlation of objective cognitive impairment in HNC survivors with subjective cognitive, physical, and psychological complaints and neurovascular abnormalities. We previously showed that lower cognitive performance correlated with subjective memory complaints and fatigue in HNC survivors > 5 years after radiotherapy [17]. This emphasizes the importance to consider both endpoints.

Besides, most studies have been performed in older patients (> 50 years) with typical, lifestyle-induced HNC. As HNC and cognitive decline may have a common etiology, such as alcohol and tobacco use [20, 21], the incidence and pathophysiology of cognitive impairment in a younger population with fewer comorbidities could differ. While research in non-HNC cancers showed that young cancer survivors may be more resilient to cognitive impairment [22], they are also in a life-stage in which they are highly active socially, professionally, and/or academically. This may explain why cognitive complaints and psychological distress are frequently reported by these patients [23]. With a rising incidence of HNC in a younger population related to the human papillomavirus (HPV) epidemic [ 24], increased research and clinical attention to cognitive impairment in young survivors are needed.

Here, we aim to estimate the association of radiotherapy of the neck with long-term cognitive functioning, fatigue, depressive symptoms, and quality of life in a unique cohort of young to middle-aged adults treated with unilateral radiotherapy of the neck. Assuming that vascular changes underlie cognitive decline, the extent and aspect of white matter hyperintensities (WMH) were related to the irradiated side of the neck.

## Methods

This study is a cross-sectional study designed to determine the long-term vascular and cognitive complications of radiotherapy of the neck in young to middle-aged adults. Participants were recruited from the Radboud university medical center, the Netherlands, and identified by querying unilateral radiotherapy of the neck between 2010 and 2015 in the radiotherapy database. Patients were eligible for inclusion when they had been treated with unilateral radiotherapy for a malignant or benign head and neck neoplasm (HNN) ≥ 5 years before inclusion. The carotid artery had to be, at least partly, in the radiotherapy target volume. Age at diagnosis had to be between 18 and 40. Exclusion criteria were contraindications to magnetic resonance imaging (MRI) and insufficient command of the Dutch language. This study was performed in line with the principles of the 1964 Declaration of Helsinki. The study was approved by the Medical Ethics Review Committee Arnhem-Nijmegen (NL71550.091.19) on November 25, 2019, and registered at clinicaltrials.gov (NCT04257968). All participants provided written informed consent.

### Radiation treatment

Before this study, all patients had been treated with external beam radiotherapy with a total dose of 26–100 Gy in 2-Gy fractions. Radiotherapy was applied with a linear accelerator (6-MV photon beams) using a three-dimensional conformal or intensity-modulated radiotherapy technique. Radiotherapy targets were defined by computed tomography and included the primary tumor site, usually the parotid gland or unilateral oropharynx, with or without the ipsilateral neck. Nobody received whole-brain radiotherapy.

### Cardiovascular risk profile

Patients were invited to the outpatient clinic of the Radboudumc Center of Expertise for Cancer Survivorship. There, a structured cardiovascular disease (CVD) risk profile was assessed based on the European guidelines on cardiovascular disease prevention [25], including the following risk factors: I) Smoking: current/former, expressed in pack-years; II) Family history of CVD: first-degree males ≤ 55 years or females ≤ 65 years with CVD; III) Hypertension: systolic blood pressure > 140 mmHg or antihypertensive medication use; IV) Diabetes mellitus: non-fasting serum glucose > 11.1 mmol/L or antidiabetic drug use; V) Hypercholesterolemia: serum low-density lipoprotein ≥ 2.6 mmol/L and/or non-high-density lipoprotein ≥ 3.4 mmol/L; VI) Overweight: body mass index ≥ 25 and/or abdominal circumference ≥ 88 cm for women or ≥ 102 cm for men; VII) Chronic daily stress: daily stress > 6 months (work, private, etcetera); and VIII) Chronic renal insufficiency: CKD-EGFR < 90 ml/min/1.73 m2 and/or albumin-to-creatinine ratio > 3.

### Neuropsychological assessment (NPA)

Education level was classified using seven categories based on the Dutch educational system, ranging from one (less than primary school) to seven (academic degree) [26]. NPAs were carried out by a trained examiner and consisted of validated, widely used tests to determine each individual’s performance in five cognitive domains: I) Episodic Memory (anterograde verbal memory), assessed by the total number correct (trials 1 to 3) and the delayed recall score of the Hopkins Verbal Learning Test [27]; II) Working Memory, measured by the backward and forward Digit Span test [28]; III) Executive Functioning, indexed by the Trail Making Test B/A ratio score [29], the Brixton Spatial Anticipation Test [30], and the Interference score of the Stroop Color-Word Test [31]; IV) Verbal Fluency (letter/category fluency) [32]; and V) Speed of Information Processing, based on the Stroop test (parts I and II) [31], Trail Making Test A [29], and Symbol Digit Substitution Test [33]. A more elaborate overview of the cognitive domain tests is provided in Appendix  1.

### Psychological questionnaires

Psychological symptoms were assessed using the Brief Symptom Inventory (BSI), measuring emotional distress on different dimensions including somatization, depression, and anxiety [34]. Fear of cancer recurrence and its impact on daily life were assessed using the Cancer Worry Scale (CWS) [35]. A severe level of fear of recurrence is identified by a score ≥ 14 [35]. Subjective memory complaints were assessed with the Cognitive Failure Questionnaire (CFQ) [36]. Fear and depressive symptoms were assessed using the Hospital Anxiety and Depression Scale (HADS) [37]. A score ≥ 11 on the depression or anxiety subscales is classified as abnormal [37]. Fatigue was assessed using the Checklist Individual Strength (CIS-20R) [38]. A score ≥ 35 on the CIS-20R Fatigue subscale indicates a heightened experience of fatigue [39]. Cancer-specific distress was assessed using the Impact of Event Scale-Revised [40]. Self-perceived speech function was assessed using the Speech Handicap Index (SHI) [41], differentiating speech function from psychosocial functioning. A score ≥ 6 identifies patients with speech problems in daily life [41]. Quality of life was assessed using the European Organization for Research and Treatment for Cancer Quality of Life Questionnaire (EORTC-QLQ-C30) [42] with functional, symptom, and global subscales.

### Brain MRI

Brain MRIs were acquired using a 3.0 Tesla MR-system (Skyra, Siemens Erlangen). The scan protocol included three-dimensional T1 magnetization-prepared rapid acquisition gradient echo (3DT1-MPRAGE), T2 turbo spin-echo (T2-TSE), and 3D fluid-attenuated inversion recovery (3DT2-FLAIR) brain sequences. Scan parameters included: 3DT1-MPRAGE) repetition time (TR)/echo time (TE) 2300/2.32 ms, inversion time (TI) 900 ms, slice thickness 0.9 mm, flip angle 8°, voxel size 0.9 × 0.9 × 0.9 mm^3^; T2-TSE) TR/TE 3500/92 ms, slice thickness 5 mm, flip angle 120°, voxel size 0.5 × 0.5 × 5 mm^3^; 3DT2-FLAIR) TR/TE 5000/394 ms, TI 1800 ms, slice thickness 1 mm, voxel size 0.5 × 0.5 × 1 mm^3^. Two experienced neuroradiologists rated brain infarctions and vascular WMH using the Fazekas scale [43]. Complementary, WMH were manually segmented on the FLAIR sequence images using Pinnacle treatment planning software (Philips Radiation Oncology Systems, Fitchburg, WI, USA) to assess WMH volumes in the irradiated and non-irradiated side. T1- and T2-weighted image sequences were used to identify WMH based on the STandards for ReportIng Vascular changes on nEuroimaging [44]. A previously published Brain Extraction Tool [45] was applied to automatically segment the T1-weighted images into brain and non-brain structures. Brain structures were subsequently segmented into grey matter (GM), white matter (WM), and cerebrospinal fluid (CSF) using the FMRIB’s Automated Segmentation Tool [46]. Tissue volumes were calculated by summing all voxel volumes belonging to that tissue type. The brain parenchymal fraction (BPF), as a measure of brain atrophy, was calculated as (volume GM + WM)/(volume WM + GM + CSF) [47].

### Statistical analysis

Cognitive performance and questionnaire scores for each individual were adjusted for age, sex, and education level using available Dutch normative data and converted into standardized Z-scores. Composite scores per cognitive domain were calculated as the average of the Z-scores of the associated subtests (in case of more than one outcome measure). A composite Z-score ≤ -1.65 (i.e. ≤ 5^th^ percentile) was classified as clinically impaired [48]. The number of patients with a composite score ≤ -1.65 or, if existing, exceeding a pre-specified cut-off value (for the psychological questionnaires) per cognitive domain and questionnaire were quantified.

Given the relatively small sample, data were expressed using the median and interquartile range (IQR) and statistical analysis was performed using non-parametric tests. One-sample Wilcoxon signed-rank tests were used to compare median Z-scores per cognitive domain and total Z-scores per questionnaire with the normative standardized median (Z = 0). In case the Wilcoxon signed-rank test was statistically significant for the total questionnaire score, individual questionnaire domains were tested accordingly. To assess the pathophysiological mechanism underlying cognitive impairments, MRI results and psychological questionnaires substantially differing from the normative mean were compared between patients with an impairment (Z-score ≤ -1.65) in ≥ 1 cognitive domain and those without such impairment using Mann–Whitney U tests. Analyses were corrected for multiple comparisons by the Benjamini–Hochberg method [49] with a false discovery rate of 0.05. Non-adjusted p-values are reported.

## Results

**Fig. 1:**
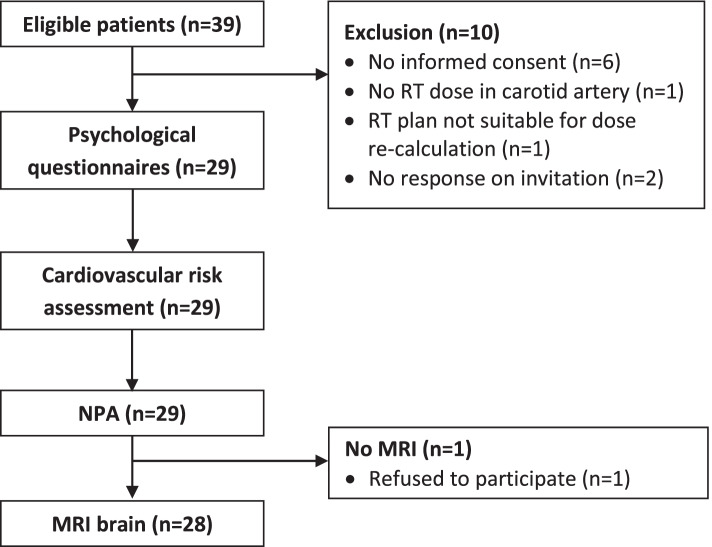
Flow-chart of patient inclusion. *NPA* = neuropsychological assessment, *RT* = radiotherapy, *MRI* = magnetic resonance imaging

Twenty-nine HNN patients were enrolled in this study. A flowchart of the patient inclusion is shown in Fig. 1. Ten additional eligible patients were excluded due to reasons stated in Fig. 1. All patients fulfilled the study protocol, except for one patient who refused a brain MRI. Patient characteristics are summarized in Table 1. Median age at inclusion was 41.0 [IQR: 33.5–47.0] years. Median follow-up after radiotherapy was 9.2 [7.3–12.9] years. One patient received a re-irradiation for a recurrence of the primary tumor resulting in a maximum physical dose of 100 Gy. Ten patients (34.5%) received concomitant chemotherapy. Five patients (17.2%) underwent extensive surgical interventions of the head and neck including a tongue (edge) resection, hemi-mandibulectomy, or cheek resection. Most patients (89.7%) had at least one CVD risk factor, including mostly hypercholesterolemia, chronic daily stress, and overweight. Cholesterol levels and renal function were unknown in 6 and 3 patients, respectively.

**Table 1:** Patient characteristics

Characteristic	***n* = 29**
**Demographics**	
Men, n (%)	13 (44.8)
Median age, years [IQR]	41.0 [33.5—47.0]
Median age at treatment, years [IQR]	35.0 [23.5—39.5]
Median follow-up after RT, years [IQR]	9.2 [7.3—12.9]
**Diagnosis, n (%)**	
Carcinoma of parotid	7 (24.1)
Pleiomorphic adenoma of parotid	8 (27.5)
Carcinoma of oropharynx	2 (6.9)
Lymphoma (primary)	8 (27.6)
Others ^a^	4 (13.8)
**Radiotherapy**	
Total dose, Gy (median [IQR], min–max)	50 [37.0—66.0], 26—100 ^b^
**Chemotherapy, n (%)**	
Total	10 (34.5)
Anthracycline	9 (31.0)
Platinum-based	2 (6.9)
Alkylating	8 (27.5)
Others	8 (27.5)
**Surgery**	
(Partial) parotidectomy	16 (55.2)
Cervical lymph node dissection	5 (17.2)
Resection tongue(edge)	2 (6.9)
(Hemi)mandibulectomy	2 (6.9)
Others	4 (13.8)
**Tumor laterality, n (%)**	
Left	18 (62.1)
Right	11 (37.9)
**CVD risk factors, n (%)**	
Smoking	
Current	6 (20.7)
Former	5 (17.2)
Pack years, median [IQR]	15.0 [5.0—25.0]
Positive family history of CVD	8 (27.6)
Chronic daily stress	10 (34.5)
Hypertension	8 (27.6)
Hypercholesterolemia	10 (43.5)
Overweight	12 (41.4)
Diabetes	1 (3.4)
Chronic renal insufficiency	3 (11.5)
**Number of CVD risk factors, n (%)**	
0	3 (10.3)
1	6 (20.7)
2	11 (37.9)
≥ 3	9 (31.0)
**Education level, median [I**Q**R]**	6.0 [5.0—7.0]
**Manual laterality**	
Right	23 (79.3)
Left	5 (17.2)
Bimanual	1 (3.4)

^a^ i.e. multiple glomus tumors, supraclavicular epithelioid hemangioendothelioma, mucoepidermoid carcinoma accessory salivary gland, and submandibular squamous cell carcinoma. ^b^ One patient underwent a re-irradiation for recurrence of the primary tumor resulting in a total radiation dose of 100 Gy. *CVD*  Cardiovascular disease

**Table 2:** Results cognitive domain data

Characteristic	Median [IQR]^a^	*P*-value^b^	Impaired, n (%)^d^
Episodic memory	-1.16 [-1.58—0.34]	< 0.001 ^c^	5 (17.2)
Working memory	-0.00 [-0.67—0.84]	0.76	4 (13.8)
Executive functioning	-0.01 [-0.26—0.42]	0.72	0 (0.0)
Verbal fluency	0.25 [-0.30—1.05]	0.07	0 (0.0)
Speed of information processing	-0.27 [-1.22—0.43]	0.14	4 (13.8)

^a^ Data of composed Z-scores are expressed as median [interquartile range] based on normative data with a mean Z-score of zero; ^b^ Not Benjamini–Hochberg adjusted p-values are shown; ^c^ Statistically significant change compared to normative data after Benjamini–Hochberg correction; ^d^ Impaired composed Z-score is defined as Z ≤ -1.65 (performance worse than 95% of the norm population)

**Table 3:** Results psychological questionnaires

Characteristic	Median [IQR]^a^	*P*-value^b^	N (%) impaired^d^
**BSI**			
Depression	-0.39 [-0.79—0.41]		4 (13.8)
Somatization	-0.72 [-0.83—0.39]		4 (13.8)
Fear	-0.69 [-0.80—0.16]		5 (17.2)
Total	-0.64 [-0.86—0.32]	0.28	5 (17.2)
**CWS**	-0.10 [-0.87—0.67]	0.91	13 (44.8)^e^
**CFQ**			
Absentmindedness	-0.32 [-1.27—1.15]		3 (10.3)
Social	-0.38 [-1.63—0.54]		1 (3.4)
Names	0.05 [-0.76—1.00]		2 (6.9)
Orientation	-0.61 [-1.63—0.21]		4 (13.8)
Total	-0.25 [-1.53—0.96]	0.24	3 (10.3)
**HADS**			
Depression	0.18 [-0.73—0.79]		2 (6.9)^e^
Anxiety	-0.86 [-1.14—0.03]		3 (10.3)^e^
Total	-0.22 [-0.86—0.26]	0.26	3 (10.3)
**CIS-20R**			
Fatigue severity	1.75 [1.21—2.00]	< 0.001^c^	15 (51.7)^e^
Concentration problems	2.30 [1.70—2.60]	< 0.001^c^	25 (86.2)
Reduced motivation	1.24 [0.76—1.98]	< 0.001^c^	9 (31.0)
Activity	1.64 [1.09—1.98]	< 0.001^c^	14 (48.3)
Total	1.94 [1.74—2.30]	< 0.001^c^	23 (79.3)
**Impact of Event Scale**			
Reliving	-0.33 [-0.55—0.05]		0 (0.0)
Avoidance	0.05 [0.05—0.59]		4 (13.8)
Total	-0.18 [-0.31—0.39]	0.96	1 (3.4)
**SHI**			
Psychosocial function	-0.24 [-0.24—1.87]		7 (24.1)
Speech function	-0.19 [-0.50—3.53]	0.03	12 (41.4)
Total	-0.23 [-0.43—3.19]		13 (44.8)^e^
**EORTC QLQ C30**			
*- Functional scales*			
Physical	0.57 [-0.46—0.65]		2 (6.9)
Role	0.50 [-0.92—0.59]		2 (6.9)
Emotional	0.04 [-0.76—0.60]		3 (10.3)
Cognitive	0.50 [-1.58—0.50]		5 (17.2)
Social	0.36 [-0.69—0.39]		3 (10.3)
Total	-0.02 [-0.70—0.51]	0.55	2 (6.9)
*- Symptom scales*			
Fatigue	0.11 [-0.79—1.16]		5 (17.2)
Nausea	-0.30 [-0.30—0.25]		0 (0.0)
Pain	-0.60 [-0.75—0.64]		4 (13.8)
Dyspnea	-0.42 [-0.42—0.39]		2 (6.9)
Sleeplessness	-0.55 [-0.65—0.63]		3 (10.3)
Eating	-0.31 [-0.31—0.23]		4 (13.8)
Constipation	-0.28 [-0.38—0.28]		2 (6.9)
Diarrhea	-0.27 [-0.32—0.27]		3 (10.3)
Financial	-0.28 [-0.28—0.23]		2 (6.9)
Total	-0.10 [-0.36—0.25]	0.60	1 (3.4)
*- Global*	0.35 [-0.11—1.05]	0.07	2 (6.9)

^a^ Data are expressed as median [interquartile-range] based on normative data with a mean Z-score of zero. Impaired Z-score is defined as Z ≤ -1.65 (performance worse than 95% of the norm population); ^b^ Not Benjamini–Hochberg adjusted p-values are shown; ^c^ Statistically significant change compared to normative data after Benjamini–Hochberg correction; ^d^ based on Z-score ≥ 1.65 or, if marked with ^e^, on a predefined clinical cut-off value (i.e. BSI-total: ≥ 14, HADS-depression ≥ 11, HADS-anxiety ≥ 11,CIS-20R fatigue severity ≥ 35, and SHI-total ≥ 6). *BSI*  Brief Symptom Inventory, *CWS *Cancer Worry Scale, *CFQ *Cognitive Failure Questionnaire, *HADS*  Hospital Anxiety and Depression Scale  *CIS-20R*  Checklist Individual Strength-20R, *SHI*  Speech Handicap Index, *EORTC QLQ C30*  European Organization for Research and Treatment for Cancer Quality of Life Questionnaire

**Table 4:** Association of cognitive impairment with fatigue, speech problems, vascular white matter hyper-intensities, and brain parenchymal fraction

Characteristic	Cognitive impairment (*n* = 10), median [IQR]	No cognitive impairment (*n* = 19), median [IQR]	P-value^a^
Fatigue (CIS-20R, Z-score)	1.74 [1.50—2.43]	1.99 [1.79—2.25]	0.70
Speech problems (SHI, Z-score)	1.59 [-0.43—4.20]	-0.43 [-0.43—2.39]	0.13
WMH, mL	0.15 [0.00—0.39]	0.09 [0.00—0.51]	0.70
BPF	0.79 [0.77—0.79]	0.78 [0.77—0.78]	0.21

^a^ Not Benjamini–Hochberg adjusted p-values are shown. *CIS-20R *Checklist Individual Strength-20R, *SHI*  Speech Handicap Index, *WMH*  white matter hyperintensities, *BPF*  brain parenchymal fraction

### Neuropsychological assessment

The neuropsychological assessment revealed a statistically significantly worse episodic-memory performance compared to normative data (Z-score = -1.16 [-1.58–0.34], *p* < 0.001) (Table 2).

### Psychological questionnaires

Forty-five percent of patients reported a severe level of fear of tumor recurrence (CWS-score ≥ 14) (Table 3). Furthermore, fatigue was highly prevalent: 51.7% experienced severe fatigue (subjective fatigue score ≥ 35) and Z-scores were statistically significant higher in all fatigue dimensions compared to normative data (*p* < 0.001). Additionally, 44.8% of patients reported speech problems in daily life (SHI-score ≥ 6). All other questionnaires did not differ from normative means.

### Brain MRI

The twenty-eight brain MRIs showed 3 brain infarctions in 2 patients (bilaterally in one patient and ipsilateral to the irradiated side in the other patient). WMH were classified as Fazekas 0 and 1 in 10 (35.7%) and 18 patients (64.2%), respectively. No patients had Fazekas 2–3 WMH. Total volume of WMH was 0.11 [0.00–0.40] mL, unrelated to radiation side (irradiated side: 0.05 [0.00–0.19] mL, control side: 0.03 [0.00–0.26] mL, *p* = 0.62). Median BPF, as a measure of brain atrophy, was 0.78 [0.77–0.79].

### Association cognitive performance, fatigue, speech problems, WMH, and brain atrophy

Patients with an impairment in ≥ 1 cognitive domain did not show statistically significant differences in fatigue severity, speech handicap index, WMH volume, or brain atrophy compared to patients without cognitive impairment (Table 4).

## Discussion

In this cross-sectional study, a unique cohort of twenty-nine young to middle-aged adult HNN survivors was included with median 9 years of follow-up after unilateral radiation of the neck. To assess long-term cognitive and psychological side-effects of HNN treatment, all patients underwent a neuropsychological assessment, completed psychological questionnaires, and had a brain MRI. HNN patients had an impaired episodic memory compared to normative data. This impairment was not associated with WMH, brain atrophy, fatigue, or self-perceived speech problems. Besides, patients reported more fatigue, fear of tumor recurrence, and speech problems compared to normative data.

With a growing, younger population of HNC survivors, partly due to the HPV epidemic, attention and prevention of long-term cognitive side-effects of treatment become increasingly important. Only limited data are available on the long-term side-effects. Most prior studies are limited to older, typical HNC patients. As a result, evidence-based personalized screening and prevention guidelines for long-term complications are lacking in clinical practice.

The memory impairment in our sample of HNN survivors many years after radiotherapy is in concordance with previous studies in HNC on short-term (< 2 years) [7, 9–14] and long-term (≥ 5 years) sequelae after treatment [14, 15]. Although previous studies also found a decreased speed of information processing, we only found a trend in this direction in HNN patients. This discrepancy may be due to the small number of patients or younger age of our study cohort with less cardiovascular risk factors (by themselves related to cognitive decline [50]) if compared to older, more typical HNC survivors. Multiple studies in hematologic, breast, and ovarian cancers showed that older age was associated with cognitive impairment after treatment [51]. Younger survivors may be more resilient to cognitive impairment due to a greater cognitive reserve [52], i.e. an individual’s ability to compensate for brain pathology [53]. This greater cognitive reserve in young patients may be related to higher neuroplasticity, i.e. the ability to form neural connections enabling compensatory pathways [22]. This high neuroplasticity leads to maintained neurocognitive performance and recovery from cognitive impairment [52].

The pathophysiological mechanism underlying cognitive impairment in HNC patients is not completely understood. Radiation to the carotid artery is thought to accelerate carotid atherosclerosis due to radiation-induced endothelial cell damage, fibrosis of the intima-medial layer, development of atherosclerotic plaques, and occlusion of the vasa vasorum leading to arterial wall ischemia [54]. Radiation-induced carotid vasculopathy, i.e. increased intima-media thickness and carotid artery stenosis has been shown in previous studies [55, 56]. Cerebrovascular lesions such as WMH [57] secondary to carotid vasculopathy [57] are associated with progressive cognitive decline and a two-fold dementia risk [58, 59] and are thought to underly cognitive impairment after neck irradiation. This is, in contrast to (scattered) radiation to the brain, an indirect effect. In the context of another study [submitted for publication], all patients included in this study underwent a bilateral carotid ultrasound. Ten patients (34%) had (a) carotid plaque(s), i.e. an intima-media thickness ≥ 1.5 mm, but they were not hemodynamically significant (stenosis percentage < 50%) and showed no signs of instability. These results, together with the low number of neurovascular lesions, make it unlikely that carotid pathology underlies the cognitive impairment found in the current study. However, also direct effects of radiotherapy to the brain have been reported in for example nasopharyngeal tumor patients [10] and cannot be excluded in the current study. Patients treated with radiotherapy for parotid gland tumors or high metastatic lymph nodes in the neck may have had low radiation doses to the ipsilateral temporal lobe or cerebellum, respectively. Although these effects are likely to be limited as radiation doses were low and direct effects are dose-dependent [10], some effect of this cannot be excluded. However, radiation doses to the frontal and parietal regions of the brain are negligible in our patient population.

Unique in this study was the comparison in neurovascular abnormalities between the radiated and non-irradiated vascular brain territory, excluding the influence of confounding factors. To our knowledge, only we have previously studied the relation between WMH and cognitive impairment in long-term HNC survivors [17]. In concordance with our previous findings, WMH were currently not associated with cognitive impairment. Cognitive scores were also not related to tumor laterality or to tumor laterality relative to manual laterality. However, we previously found an association between brain infarctions and cognitive impairment that was absent in the current study. The two patients with brain infarctions in the current study reported severe fatigue (Z-score = 2.05–2.84) but did not have any other cognitive or psychological impairments. Cognitive decline in HNC patients has furthermore been associated with fatigue, fear, depressive symptoms, and self-perceived speech problems [14, 17, 60]. However, we currently did not find such association. These discrepancies may be explained by different aspects of brain infarctions, e.g. localization, size, and time-since brain infarction. Alternatively, there is a common etiology of HNC and cognitive decline in older age, such as alcohol and tobacco use, that are less common in our study cohort. A different pathophysiological mechanism underlying cognitive decline in younger patients would entail alternative prevention strategies in this population.

A growing and younger population of HNC survivors also asks for adjustments of screening and prevention methods to age-specific psychosocial complaints. Fatigue is reported in one-fourth of young adult cancer survivors 5–30 years after treatment [61]. An even higher prevalence (up to 79%) was also found ≥ 5 years after treatment in older, typical HNC patients [17]. Despite its high prevalence, this symptom is underrecognized and often left untreated [62]. Fatigue may negatively influence work, social relationships, mood, daily activities, and quality of life [63]. With work and a career at stake, fatigue may have an even higher impact on younger HNC survivors. Attention to the presence and possible treatment of fatigue is, therefore, necessary in this population.

Besides fatigue, fear of cancer recurrence is one of the most important late effects in HNC survivors [64]. While the likelihood of recurrence is highest within two years after diagnosis [65], the intensity of fear of recurrence does not significantly change over time [66, 67]. High fear of tumor recurrence is reported in 31–52% of patients 1–20 years after treatment amongst different cancer types [35, 68–70]. Although not all tumors in our cohort were malignant, we found a comparable high rate of fear of recurrence of 44.8%. Psychological interventions including (contemporary) behavioral therapy have shown to be effective in sustainably reducing this fear and, therefore, should be considered in this population.

Speech problems can be differentiated in speech function (e.g. articulation problems due to anatomical-physiological dysfunction resulting from surgery or radiotherapy) and psychosocial function (e.g. shame of speech function). Due to the different tumor nature and treatment in HNN patients compared to HNC patients with lower radiotherapy doses, less mutilating surgeries (mostly parotidectomies), and less tumor involvement of the larynx, our cohort cannot be compared to HNC patients. Besides the abovementioned speech problems, it is also important to account for communication problems due to speech formulation and perception (e.g. word-finding difficulties due to cognitive impairment resulting from neurovascular damage) that are currently not incorporated in screening tools.

Despite the memory impairment, patients did not report cognitive complaints. Multiple studies showed that patients' subjective cognitive symptoms do not correlate with objective cognitive function [71, 72]. Additionally, QoL was relatively unaffected in HNN patients despite the high prevalence of fatigue, fear of tumor recurrence, and self-reported speech problems. In other words, psychosocial complaints minimally affect daily life in these patients. This is consistent with other studies in long-term HNC survivors [17, 73, 74]. QoL is typically most affected shortly after diagnosis but returns to baseline values 48–72 months after treatment [75]. This is presumably due to a change in internal standards, values, and priorities of patients over time, known as ‘response shift’ [76].

Some limitations of our study must be taken into consideration when interpreting the results. The small sample limits statistical analysis and inferences as possible confounders, such as cardiovascular disease, alcohol use, concomitant treatment (among others surgical intervention and chemotherapeutic treatment), and time-since-treatment could not be corrected for. These concomitant treatments are, however, known to affect cognitive performance in HNC patients [7, 8, 11, 77]. Furthermore, pre-treatment cognitive and psychosocial functioning were not assessed, while a decreased cognitive performance has been reported before treatment in HNC patients [60, 78, 79]. The lack of a control group to assess cognitive impairment could have induced false positive results to assess cognitive impairment [77]. A large prospective, longitudinal study including a control group not exposed to radiotherapy but otherwise treated similarly with similar demographic and socioeconomic status assessing pre- and posttreatment cognitive performance, and considering the time-since-treatment, would be valuable for future studies. With the inclusion of unilateral irradiated HNN patients, we could compare neurovascular abnormalities, related to cognitive impairment, in the radiated and non-irradiated vascular brain territory unaffected by possible confounding factors. However, we did not find such asymmetry. Further studies with an even longer follow-up and higher radiation doses are warranted to verify this lack of asymmetry. Additionally, the current study only focused on structural neuroimaging (WMH and infarctions). More advanced neuroimaging techniques, including diffusion-weighted imaging, susceptibility-weighted imaging, and functional MRI would be valuable to detect microstructural damage. Diffusion-weighted imaging is sensitive to white matter damage in both WMH and normal-appearing white matter [80], and diffusion tensor imaging measures have been shown to correlate more strongly with cognitive impairment than WMH [81]. In contrast to previous studies in HNC patients, we also included patients with benign HNN with possibly fewer side-effects as only including HNC patients would have resulted in a very small sample. Furthermore, patients were partly included during the COVID-19 pandemic which may have influenced patients’ psychosocial functioning, but this influence remains to be assessed.

## Conclusions

In conclusion, we evaluated long-term cognitive and psychological side-effects of head and neck radiotherapy in young to middle-aged adults. HNN patients had an episodic memory impairment that was not associated with WMH, brain atrophy, fatigue, or self-perceived speech problems. Moreover, HNN patients reported severe fatigue compared to normative data and had a high level of fear of tumor recurrence and subjective speech problems. Attention, prevention, and possible early treatment of long-term cognitive and psychosocial side-effects are warranted in HNC patients.

## Supplementary information

**Additional file 1:  Appendix 1.** Cognitive domains with corresponding subtests of the neuropsychological assessment

## Data Availability

The datasets generated during and/or analysed during the current study are available from the corresponding author on reasonable request.

## Abbreviations

BPF: Brain parenchymal fraction
BSI: Brief Symptom Inventory
CFQ: Cognitive Failure Questionnaire
CIS-20R: Checklist Individual Strength
CSF: Cerebrospinal fluid
CVD: Cardiovascular disease
CWS: Cancer Worry Scale
3DT1-MPRAGE: Three-dimensional T1 magnetization-prepared rapid acquisition gradient-echo
3DT2-FLAIR: Three-dimensional T2 fluid-attenuated inversion recovery
EORTC-QLQ-C30: European Organization for Research and Treatment for Cancer Quality of Life Questionnaire
GM: Gray matter
HADS: Hospital Anxiety and Depression Scale
HNC: Head and neck cancer
HNN: Head and neck neoplasms
HPV: Human papillomavirus
IQR: Interquartile range
MRI: Magnetic resonance imaging
NPA: Neuropsychological assessment
SHI: Speech Handicap Index
T2-TSE: T2 turbo spin-echo
TE: Echo time
TI: Inversion time
TR: Repetition Time
WM: White matter
WMH: White matter hyperintensities
