# Supplementary material for: Long-term cognitive, psychosocial, and neurovascular complications of unilateral head and neck irradiation in young to middle-aged adults
Source: BMC Cancer. 2022 Mar 5;22:244. doi: 10.1186/s12885-022-09295-9 (PMC8897732; doi:10.1186/s12885-022-09295-9)
Supplement: Supplementary file 1 — Additional file 1: Appendix 1. Cognitive domains with corresponding subtests of the neuropsychological assessment [file 12885_2022_9295_MOESM1_ESM.docx]

| Appendix 1: Cognitive domains with corresponding subtests of the neuropsychological assessment | | | |  |
| --- | --- | --- | --- | --- |
| Cognitive domain | **Test(s)** | **Description** | **Measure** | |
| Episodic memory | Hopkins Verbal Learning Test (Dutch version 5-trail Rey Auditory Verbal Learning) [21] | Number of five words recalled immediately (immediate recall) and after a delay (delayed recall) | Ability to acquire and retain new verbal information | |
| Working memory | WAIS-IV Digits Span test Backwards + Forwards [22] | Repeat sequences of digits in same (forwards) or reversed (backwards) order | Working memory | |
| Executive Functioning | Trail Making Test [23] | Connect numbers (1-25) in ascending order (part A) or numbers alternated with letters (part B), i.e. 1-A-2-B, etc. | Cognitive flexibility | |
|  | Brixton Spatial Anticipation [24] | Visuospatial sequencing task with rule changes | Rule detection & set shifting | |
|  | Interference score of the Stroop color-word test [25] | Seconds needed to read “word card” (color-words in black)/mean duration to read “color” (colored patches that need to be named) and color-word card (color-names printed in incongruent colors that need to be read) | Response inhibition | |
| Verbal fluency | Fluency letter (D-A-T) and  Fluency animal naming [26] | Name as many words beginning with a certain letter <60s  Name as many animals <60s | Letter fluency  Semantic fluency | |
| Speed of information processing | Symbol Digit substitution task [27] | 9 digit-symbol pairs (e.g. 1/-,2/┴) followed by randomly ordered digits where corresponding symbols need to be drawn within 90s | Processing speed, working memory, visuospatial processing, and attention | |
|  | Trail Making test A [23] | Connect numbers (1-25) in ascending order (part A) or numbers alternated with letters (part B), i.e. 1-A-2-B, etc. | Attention, visual search and motor function | |
|  | Mean score part I and II of Stroop test [25] | Mean duration to read “word card” (color-words in black) and “color card” (colored patches that need to be named) | Processing speed | |
